# Lab on a chip genotyping for *Brucella *spp. based on 15-loci multi locus VNTR analysis

**DOI:** 10.1186/1471-2180-9-66

**Published:** 2009-04-07

**Authors:** Riccardo De Santis, Andrea Ciammaruconi, Giovanni Faggioni, Raffaele D'Amelio, Cinzia Marianelli, Florigio Lista

**Affiliations:** 1 Sezione di Istologia e Biologia molecolare, Centro Studi e Ricerche di Sanità e Veterinaria Militare, Via Santo Stefano Rotondo 4, 00184 Rome, Italy; 2 Cattedra di Allergologia e Immunologia Clinica, II Facoltà di Medicina, Università di Roma "La Sapienza", Rome, Italy; 3 Dipartimento di Sanità Alimentare ed Animale, Istituto Superiore di Sanità, Viale Regina Elena 299, 00161 Rome, Italy

## Abstract

**Background:**

Brucellosis is an important zoonosis caused by the genus *Brucella*. In addition *Brucella *represents potential biological warfare agents due to the high contagious rates for humans and animals. Therefore, the strain typing epidemiological tool may be crucial for tracing back source of infection in outbreaks and discriminating naturally occurring outbreaks versus bioterroristic event. A Multiple Locus Variable-number tandem repeats (VNTR) Analysis (MLVA) assay based on 15 polymorphic markers was previously described. The obtained MLVA band profiles may be resolved by techniques ranging from low cost manual agarose gels to the more expensive capillary electrophoresis sequencing. In this paper a rapid, accurate and reproducible system, based on the Lab on a chip technology was set up for *Brucella *spp. genotyping.

**Results:**

Seventeen DNA samples of *Brucella *strains isolated in Sicily, previously genotyped, and twelve DNA samples, provided by MLVA *Brucella *VNTR ring trial, were analyzed by MLVA-15 on Agilent 2100. The DNA fragment sizes produced by Agilent, compared with those expected, showed discrepancies; therefore, in order to assign the correct alleles to the Agilent DNA fragment sizes, a conversion table was produced. In order to validate the system twelve unknown DNA samples were analyzed by this method obtaining a full concordance with the VNTR ring trial results.

**Conclusion:**

In this paper we described a rapid and specific detection method for the characterization of *Brucella *isolates. The comparison of the MLVA typing data produced by Agilent system with the data obtained by standard sequencing or ethidium bromide slab gel electrophoresis showed a general concordance of the results. Therefore this platform represents a fair compromise among costs, speed and specificity compared to any conventional molecular typing technique.

## Background

The genus *Brucella *comprises Gram-negative bacteria that are the etiological agents of brucellosis, a zoonosis that represents one of the most important worldwide disease affecting animals and humans, especially in the Mediterranean areas [1,2]. The disease can be transmitted to humans directly by contact with infected animals or indirectly by contaminated dairy products. *Brucella *infections can cause chronic debilitating diseases in humans with the involvement of multiple organs and low mortality while in the domesticated animals can lead to reproductive failure with subsequent economic loss. Six classically species are recognized within the genus *Brucella*: *Brucella abortus *(7 biovars), *Brucella melitensis *(3 biovars), *Brucella suis *(5 biovars), *Brucella ovis*, *Brucella canis*, and *Brucella neotomae *[3]. Isolation and characterization of novel different *Brucella *strains from a wide variety wildlife species from terrestrial and marine mammals, recently classified as three additional species, *B. ceti *(cetaceans) *B. pinnipedialis *and *B. microti *underline the importance of wildlife as reservoir for zoonotic brucellosis [4,5]. In addition, *Brucella *spp. represent potential biological warfare agents due to the high contagious rates for humans and animals [6,7]. Therefore it is very important to have a strain typing epidemiological tool for source trace back in outbreaks of infection. Characterization of *Brucella *at species and biovar level can be performed by differential microbiological approaches used for phenotyping [8]. However, these biotyping methods are time consuming and potentially hazardous for laboratory personnel. In addition, the limited variation among some species and biovars may result in conflicting data and complicate interpretation [9,1]. Biotyping methods are not therefore suitable for epidemiological tracking where a more accurate identification is necessary. Thus, genetic characterization using molecular DNA technology has been investigated and several molecular techniques for subtyping have been proposed. In the last years PCR-based assays have been employed in an attempt to find suitable DNA markers for the molecular typing of *Brucella*. Examples are repetitive intergenic palindromic sequence-PCR (REP-PCR) [10], random amplified polymorphic DNA-PCR (RAPD-PCR) [11], arbitrary primed-PCR (AP-PCR) [12], amplified fragment length polymorphism (AFLP) [13], single nucleotide polymorphism (SNP) [14,15], and polymerase chain reaction-restriction fragment length polymorphism (PCR-RFLP) [16]. Recently, a highly discriminatory typing system called MLVA, based on multilocus characterization of variable number of tandem repeats (VNTRs), has been proposed for several pathogens such as *Haemophilus influenzae *[17], *Mycobacterium tuberculosis *[18], *Bacillus anthracis *and *Yersinia pestis *[19-21]. The multilocus VNTR analysis methods (MLVA-15 and -16) were also described for *Brucella *genotyping [22,23,6]. The MLVA-15 is based on 15 polymorphic markers subdivided in two panels: the panel 1, consisting of 8 minisatellite markers with a good species identification capability, and the panel 2, consisting of 7 microsatellite markers with higher discriminatory power. MLVA-15 assay generates multiple band profiles or fingerprint patterns and represents a useful molecular approach for *Brucella *typing in spite of the high DNA homology among *Brucella *species (90%) [24]. The obtained MLVA band profiles may be resolved by different techniques ranging from low cost manual agarose gels to the more expensive capillary electrophoresis sequencing systems. Recently, a rapid and inexpensive method based on the Lab on a chip technology (Agilent Technologies) has been proposed. This miniaturized platform for electrophoresis applications is able to size and quantitate PCR fragments, and was previously used for studying the genetic variability of bcIA gene of *Bacillus cereus *[25] and for genotyping of *Bacillus anthracis *and *Yersinia pestis *[26]. In this paper we evaluate the possibility to use the Agilent 2100 Bioanalyzer equipment for MLVA typing of *Brucella *strains using the selected subset of 15 loci proposed by La Flèche [23]. On that account, we analyzed by Agilent technology the collection of seventeen human *Brucella *isolates whose MLVA fingerprinting profiles were previously resolved by capillary electrophoresis sequencing system [27] and results compared, while twelve DNA samples, provided in 2007 for a MLVA VNTR ring trial, were *de novo *genotyped.

## Results

In order to set up this method, we analyzed, by MLVA-15 [23] on Agilent 2100 Bioanalyzer, seventeen *Brucella *strains previously identified as *B. melitensis *biovar 3 by direct sequencing [27]. The 15 VNTR markers amplified for bacterial genotyping were arranged into three duplex and nine singleplex PCR reactions. The arrangement of different loci in the same multiplex was such to avoid overlapping of VNTR markers size ranges; in this way it was possible to use a single 12-wells chip (DNA 1000 LabChip Kit) for strain analysis. After PCR amplification 1 μl of each reaction was loaded into the chip and the amplification product size estimates were obtained by the Agilent Software. These values showed discrepancies compared with those expected [27], making difficult the allele assignment directly from rough data. In order to assign the correct alleles to the Agilent DNA fragment sizes, a conversion table containing for each locus the expected size, the range of observed sizes, including arithmetical average ± standard deviation, and the corresponding allele was produced (Additional File 1). We could establish experimentally the variability range for each allele. Even if we didn't conduct an extensive study on migration, these ranges were determined considering interchip/intrachip variability from the same amplification product or different amplification of the same strain allele or, for the same alleles, different strain amplification (data not shown). The data are considered valuable only if standard deviation is lower than the 50% of the repeat unit length. In this way, we could measure a variation proportionated to the relative number of nucleotides in each repeat unit. All the allele measurements satisfied this criterion, allowing the unambiguous assignment of the correct allele to each observed value (Additional File 1). In order to validate this platform, we analyzed twelve unknown samples provided by Dr. Falk Melzer for MLVA Brucella 2007 ring trial [28]. The Agilent 2100 Bioanalyzer MLVA-15 products were separated and DNA fragment sizes were correlated to the alleles by the conversion table. The resulting fingerprint for each strain was matched against the MLVA database Brucella test [29], allowing identification of samples and their genetic relationship with the other database strains (Table 1). The identified species were compared with the VNTR ring trial results [28], obtaining a full concordance.

**Table 1:** The twelve strains provided for the Ring trial Brucella 2007.

Strains^a^	Species	Biovar	Classification according MLVA Database Genotyping^b^	Origin
bru015	B. suis	2	Thomsen (ATCC23445; BCCN R13)	Denmark
bru002	B. abortus	1	544 (ATCC 23448; BCCN R4)	England
bru011	B. melitensis	2	63/9 (ATTC 23457; BCCN R2)	Turkey
bru004	B. abortus	3	Tulya (ATCC 23450; BCCN R6)	Uganda
Bru517/bru522	B. canis		B. canis	Romania/France
bru016	B. suis	3	686 (ATCC 23446; BCCN R14)	United States
bru009	B. melitensis	3	Ether (ATCC 23458; BCCN R3)	Italia
bru003	B. abortus	2	86/8/59 (ATCC 23449; BCCN R5)	England
bru537	B. ovis			France (64)
bru022	B. pinnipediae		B2/94 (BCCN 94–73)	Scotland
bru014	B. suis	1	1330 (ATCC 23444; BCCN R12)	United States
bru001	B. melitensis	1	16M (ATTC 23456; BCCN R1)	United States

^a ^Strains according to Le Flèche [23]

^b ^MLVA bank for bacterial genotyping [29]

## Discussion

The renewed threat of biological weapons and the appearance of zoonotic infections caused by *Brucella *spp. have reinforced the need to provide a rapid and specific detection method for the characterization of *Brucella *isolates. In fact, the discrimination between natural outbreaks and/or intentional release of micro-organism agents may be of crucial importance in the context of the bioterrorism. *Brucella *strains differentiation in the last years was obtained by assays based on the analysis of the polymorphism of tandemly repeated DNA sequences. This strategy has proven to be appropriate for pathogenic bacterial species typing with a high genetic homogeneity. Recently a new multilocus VNTR analysis (MLVA-15) method, based on a subset of fifteen tandem repeat loci, was described demonstrating high discriminatory power [23]. The different alleles, amplified by standard PCR techniques, can be analyzed by several electrophoretic techniques that can be time-consuming as agarose gel, or require high costs for reagents and instruments as capillary electrophoresis sequencing. Our attention was addressed on the Agilent 2100 "Lab on a Chip" platform, in order to obtain a more rapid and inexpensive method of discrimination. The loading of the fifteen amplification products in a single chip provides an increased time-reduction (chip run time is approximately 30 minutes) as compared to assay run on agarose gels for the same analysed markers. After data collection and analysis, we observed that the size estimated by the Agilent 2100 Bioanalyzer were shifted by a variable value (offset) in respect to the real size. Indeed, the estimated sizes were shifted of a variable value (offset) respect to the real size estimated by sequencing and each offset showed a variable range. These differences could be due to the different nature of the gel matrix or to a slightly biased flanking sequence or repeat unit specific mobility pattern [21]. These discrepancies between observed and expected sizes have been overcome creating a correspondence table which allows for each observed value to assign the expected size and corresponding allele (Additional File 1). The comparison of the results obtained by the multilocus VNTR analysis (MLVA-15) method [23] on the Agilent 2100 "Lab on a Chip" platform showed a good size correlation with nucleotide sequence analysis, confirming the rapidity and efficiency of this platform respect to standard sequencing or ethidium bromide slab gel electrophoresis. Furthermore, the possibility to compare different chip data in different times made the system suitable for epidemiological purposes. We consider this system one the most promising platforms for MLVA-15 typing because offers a fair compromise among costs, speed and specificity compared to any of the conventional molecular typing techniques.

## Conclusion

In this paper is described a rapid, accurate and reproducible system for genotyping of *Brucella*. The method is based on Multiple Locus Variable Number Tandem Repeats (VNTR) Analysis (MLVA) assay with 15 markers (MLVA-15), previously described, and the Agilent 2100 Bioanalyzer, for the separation of nucleic acid molecules. The validation of the system was performed reanalyzing seventeen DNA samples, previously genotyped by traditional techniques, and twelve DNA provided for a VNTR ring trials, obtaining a general concordance of the results. Therefore MLVA typing data produced by Agilent system represents an alternative to standard sequencing or ethidium bromide slab gel electrophoresis.

## Methods

### Brucella strains and DNA extraction

In this study, seventeen *Brucella *strains isolate from Sicilian hospitalized patients with acute brucellosis [27], and twelve DNA samples, provided by Dr. Falk Melzer for the Ring trial Brucella 2007, were analysed. DNA was extracted using proteinase K and sodium dodecyl sulfate method. Pellets were resuspended in 50 μl of nuclease-free water. Twenty nanograms of DNA template were used for PCR amplifications.

### VNTR amplification

VNTR amplifications were performed according to Le Flèche et al [23]. Fifteen sets of primers previously proposed were used: Bruce06, Bruce08, Bruce11, Bruce12, Bruce42, Bruce43, Bruce45, Bruce55 (panel 1), and Bruce04, Bruce07, Bruce09, Bruce16, Bruce18, Bruce21 and Bruce30 (panel 2). The 15 markers were arranged into 3 duplex, indicated as multiplex 1a, 2b and 3c respectively for the loci bruce 43 and bruce 08, bruce 12 and bruce 18 and bruce 11 and bruce 21 and 9 singleplex. Amplification reaction mixtures were prepared in 15 μl volumes using 1U FastStart polymerase Taq (Roche) and containing 1 ng of DNA, 1× PCR Roche reaction buffer (10 mM Tris-HCl, 2,5 mM MgCl_2_, 50 mM KCl pH 8.3), 0.2 mM dNTPs (Roche) and 0,3 μM of each flanking primer. Thermal cycling, conducted on a Peltier Thermal Cycler DNA Engine DYAD (MJ Research), was performed as follows: The optimized protocol was, after an initial heating at 95°C for 5 min, 35 cycles denaturation at 95°C for 30 sec, annealing at 60°C for 30 sec and extension at 70°C for 60 sec. A final extension was performed at 70°C for 5 min.

### MLVA-15 analysis

The amplification products were loaded into chip wells prepared according to manufacturer recommendations (DNA 1000 LabChip Kit). Each chip contains 16 wells: 12 for the samples, 3 for gel mix. After gel preparation, each sample well was loaded with 1 μl of PCR reaction and 5 μl of internal marker (containing two MW size standards of 15 and 1500 bp). One microliter of DNA ladder was loaded in the ladder well. Finally, the chip was vortexed for 60 sec and inserted into Agilent 2100 Bioanalyzer. During the separation of the fragments, the samples were analyzed sequentially and electropherograms, virtual gel images and table data were shown. Amplification product size estimates were obtained by using the Agilent 2100 Expert Software version B.02.03.SI307 firmware C.01.055 (Agilent Technologies).

## Authors' contributions

RDS and AC did the set up of the Brucella 15-MLVA assay. RDS, AC and CM participated to typing work. FL, RD'A and CM did the error checking analysis. RDS and GF did various sequence analysis. FL and RDS were in charge of the database and clustering analyses. FL, AC, RD'A and RDS conceived the study. FL and RDS wrote the report. All authors read and approved the final manuscript

## Supplementary Material

Additional file 1 **Comparison between *Brucella *product sizes inferred by Agilent 2100**. Bioanalyzer software – Observed size and their arithmetic average (x) ± standard deviation (σ) – and actual sizes obtained by direct sequencing of the PCR product or data available in Genbank (Expected size). Unit Length size (UL bps). Click here for file
