# Supplementary material for: Lab on a chip genotyping for Brucella spp. based on 15-loci multi locus VNTR analysis
Source: BMC Microbiol. 2009 Apr 7;9:66. doi: 10.1186/1471-2180-9-66 (PMC2674449; doi:10.1186/1471-2180-9-66)
Supplement: Additional file 1 — Comparison between Brucella product sizes inferred by Agilent 2100. Bioanalyzer software – Observed size and their arithmetic average (x) ± standard deviation (σ) – and actual sizes obtained by direct sequencing of the PCR product or data available in Genbank (Expected size). Unit Length size (UL bps). [file 1471-2180-9-66-S1.doc]

**Additional File 1:** Comparison between *Brucella* product sizes inferred by Agilent 2100 Bioanalyzer software - Observed size and their arithmetic average (x) ± standard deviation (σ) - and actual sizes obtained by direct sequencing of the PCR product or data available in Genbank (Expected size). Unit Length size (UL bps).

| PCR | Locus (UL bps) | Allele | Expected size | Observed size | x ±σ |
| --- | --- | --- | --- | --- | --- |
| Multiplex 1 | Bruce 08 (18) | 2 | 312 |  |  |
|  |  | 3 | 330 | 341-347 | 344.8 ±2 |
|  |  | 4 | 348 | 354-364 | 360.1 ±3.3 |
|  |  | 5 | 366 | 365-380 | 375.9 ±4.4 |
|  |  | 6 | 384 | 391-394 | 392.6 ±1.2 |
|  | Bruce 43 (12) | 1 | 170 | 170-178 | 173.2 ±1.2 |
|  |  | 2 | 182 | 185-188 | 186.4 ±1.1 |
|  |  | 3 | 194 |  |  |
| Multiplex 2 | Bruce 12 (15) |  |  |  |  |
|  |  | 7 | 302 |  |  |
|  |  | 8 | 317 |  |  |
|  |  | 9 | 332 |  |  |
|  |  | 10 | 347 | 334-344 | 340.5 ±3.6 |
|  |  | 11 | 362 | 351-357 | 354.8 ±1.9 |
|  |  | 12 | 377 | 362-373 | 367.5 ±2.8 |
|  |  | 13 | '392 | 377-389 | 383.6 ±3.4 |
|  |  | 14 | 407 | 391-399 | 396.4 ±2 |
|  |  | 15 | 422 | 400-410 | 406.2 ±3.7 |
|  |  | 16 | 437 |  |  |
|  |  | 17 | 452 |  |  |
|  | Bruce 18 (8) | 3 | 130 |  |  |
|  |  | 4 | 138 | 141-147 | 145.4 ±1.9 |
|  |  | 5 | 146 | 152-154 | 153.3 ±0.8 |
|  |  | 6 | 154 | 160-165 | 161.7 ±1.2 |
|  |  | 7 | 162 | 168-172 | 170.9 ±0.8 |
|  |  | 8 | 170 | 177-179 | 178.1 ±1.3 |
|  |  | 9 | 178 |  |  |
| Multiplex 3 | Bruce 11 (63) | 2 | 257 | 258-260 | 259.0 ±1.4 |
|  |  | 3 | 320 | 314-323 | 320.4 ±2.4 |
|  |  | 4 | 383 | 373-382 | 377.8 ±2.4 |
|  |  | 5 |  |  |  |
|  |  | 6 | 509 | 504-510 | 506.4 ±1.5 |
|  |  | 7 |  |  |  |
|  |  | 8 | 635 | 628-633 | 631 ±2 |
|  |  | 9 | 698 | 672-676 | 673.8 ±1.8 |
|  |  | 10 | 761 |  |  |
|  |  | 11 | 824 |  |  |
|  |  | 12 | 887 |  |  |
|  |  | 13 | 950 |  |  |
|  |  | 14 | 1013 |  |  |
|  |  | 15 | 1076 |  |  |

| PCR | Locus (UL bps) | Allele | Exp. size | Observed size | x ±σ |
| --- | --- | --- | --- | --- | --- |
| Multiplex 3 | Bruce 21 (8) | 5 | 140 |  |  |
|  |  | 6 | 148 | 155 |  |
|  |  | 7 | 156 | 163-166 | 164.5 ±2.1 |
|  |  | 8 | 164 | 169-173 | 171.6 ±0.9 |
|  |  | 9 | 172 | 182-184 | 182.5 ±0.6 |
| Singleplex 4 | Bruce 06 (134) | 1 | 140 | 143-149 | 146.3 ±3 |
|  |  | 2 | 274 | 276-283 | 280.8 ±1.7 |
|  |  | 3 | 408 | 405-423 | 416.9 ±4.7 |
|  |  | 4 | 542 | 559-575 | 566.50 ±8.2 |
| Singleplex 5 | Bruce 42 (125) | 1 | 164 | 162-166 | 164 ±1.1 |
|  |  | 2 | 289 | 277-284 | 281.6 ±2.1 |
|  |  | 3 | 414 | 396-402 | 397.6 ±2 |
|  |  | 4 | 539 | 527-536 | 530.6 ±3.5 |
|  |  | 5 | 664 |  |  |
|  |  | 6 | 789 | 754-767 | 759.8 ±6.7 |
|  |  | 7 | 914 |  |  |
| Singleplex 6 | Bruce 45 (18) | 2 | 133 |  |  |
|  |  | 3 | 151 | 149-154 | 152 ±1.3 |
|  |  | 4 | 169 |  |  |
|  |  | 5 | 187 | 182-187 | 185.4 ±1.4 |
|  |  |  |  |  |  |
| Singleplex 7 | Bruce 55 (40) | 1 | 193 |  |  |
|  |  | 2 | 233 | 231-243 | 239 ±3 |
|  |  | 3 | 273 | 270-283 | 278.2 ±3 |
|  |  | 4 | 313 | 315-316 | 315.5 ±0.7 |
|  |  | 5 | 353 |  |  |
|  |  | 6 | 393 |  |  |
|  |  | 7 | 433 |  |  |
| Singleplex 8 | Bruce 30 (8) | 2 | 119 | 126-127 | 126.5 ±0.7 |
|  |  | 3 | 127 | 131-138 | 135.2 ±1.5 |
|  |  | 4 | 135 | 140-145 | 142.2 ±1.9 |
|  |  | 5 | 143 | 149-153 | 150.6 ±1.5 |
|  |  | 6 | 151 | 161-162 | 161.5 ±0.7 |
|  |  | 7 | 159 |  |  |
|  |  | 8 | 167 |  |  |
|  |  | 9 | 175 |  |  |
|  |  | 10 | 183 |  |  |
|  |  | 11 | 191 |  |  |
|  |  | 12 | 199 |  |  |

| PCR | Locus (UL bps) | Allele | Expected size | Observed size | x ±σ |
| --- | --- | --- | --- | --- | --- |
| Singleplex 9 | Bruce 04 (8) | 1 | 144 |  |  |
|  |  | 2 | 152 | 161-164 | 162.5 ±2.1 |
|  |  | 3 | 160 | 169-175 | 171.6 ±2 |
|  |  | 4 | 168 | 177-182 | 179.1 ±1.3 |
|  |  | 5 | 176 | 185-191 | 187.3 ±1.8 |
|  |  | 6 | 184 | 194-198 | 195.7 ±1.3 |
|  |  | 7 | 192 | 201-207 | 203.4 ±2.2 |
|  |  | 8 | 200 | 213-214 | 213.7 ±0.6 |
|  |  | 9 | 208 | 219-222 | 220.5 ±2.1 |
|  |  | 10 | 216 |  |  |
|  |  | 11 | 224 |  |  |
|  |  | 12 | 232 |  |  |
|  |  | 13 | 240 |  |  |
|  |  | 14 | 248 |  |  |
|  |  | 15 | 256 |  |  |
|  |  | 16 | 264 |  |  |
|  |  | 17 | 272 |  |  |
|  |  | 18 | 280 |  |  |
|  |  | 19 | 288 |  |  |
|  |  | 20 | 296 |  |  |
|  |  | 21 | 304 |  |  |
|  |  | 22 | 312 |  |  |
|  |  | 23 | 320 |  |  |
|  |  | 24 |  |  |  |
|  |  | 25 |  |  |  |
|  |  | 28 | 360 |  |  |
| Singleplex 10 | Bruce 07 (8) | 2 | 134 |  |  |
|  |  | 3 | 142 |  |  |
|  |  | 4 | 150 | 150-154 | 151.9 ±1.5 |
|  |  | 5 | 158 | 157-162 | 159.8 ±1.4 |
|  |  | 6 | 166 | 166-171 | 168.1 ±1.4 |
|  |  | 7 | 174 | 175-178 | 176.8 ±1 |
|  |  | 8 | 182 | 183-186 | 184.4 ±1.1 |
|  |  | 9 | 190 | 192-195 | 195 ±1.5 |
|  |  | 10 | 198 | 200 |  |
|  |  | 11 | 206 |  |  |
|  |  | 12 | 214 |  |  |
|  |  | 13 | 222 |  |  |
|  |  | 14 | 230 |  |  |
|  |  | 15 |  |  |  |
|  |  | 16 | 246 |  |  |

| PCR | Locus (UL bps) | Allele | Expected size | Observed size | x ±σ |
| --- | --- | --- | --- | --- | --- |
| Singleplex 11 | Bruce 09 (8) | 3 | 124 | 127-132 | 129.9 ±1.8 |
|  |  | 4 | 132 | 141 |  |
|  |  | 5 | 140 | 145-147 | 146 ±1.4 |
|  |  | 6 | 148 | 152-158 | 155.2 ±1.8 |
|  |  | 7 | 156 | 161-166 | 163.5 ±1.3 |
|  |  | 8 | 164 | 171-175 | 173.2 ±1.5 |
|  |  | 9 | 172 | 180-183 | 182 ±1.3 |
|  |  | 10 | 180 | 186-190 | 188 ±1.4 |
|  |  | 11 | 188 | 195-202 | 197.7 ±3 |
|  |  | 12 | 196 | 204-208 | 205.7 ±2.1 |
|  |  | 13 | 204 | 214-218 | 216 ±2 |
|  |  | 14 | 212 |  |  |
|  |  | 15 | 220 |  |  |
|  |  | 16 | 228 |  |  |
|  |  | 17 | 236 |  |  |
|  |  | 18 | 244 | 253-260 | 256.7 ±3.3 |
|  |  | 19 | 252 |  |  |
|  |  | 20 | 260 |  |  |
|  |  | 21 | 268 |  |  |
|  |  | 22 | 276 |  |  |
|  |  | 23 | 284 |  |  |
|  |  | 24 | 292 |  |  |
| Singleplex 12 | Bruce 16 (8) | 2 | 144 | 145-149 | 147.4 ±1.3 |
|  |  | 3 | 152 | 152-158 | 154.9 ±2.1 |
|  |  | 4 | 160 | 160-167 | 163.1 ±2.5 |
|  |  | 5 | 168 | 169-177 | 173 ±2.7 |
|  |  | 6 | 176 | 179-185 | 181.9 ±2.2 |
|  |  | 7 | 184 | 187-192 | 189.2 ±2.2 |
|  |  | 8 | 192 | 196-201 | 198.2 ±2.2 |
|  |  | 9 | 200 | 203-209 | 205.4 ±2.3 |
|  |  | 10 | 208 | 212-214 | 213 ±1.4 |
|  |  | 11 | 216 | 223-231 | 225.2 ±3.3 |
|  |  | 12 | 224 |  |  |
|  |  | 13 |  |  |  |
|  |  | 14 | 240 |  |  |
|  |  | 15 | 248 |  |  |
